# Urine-Derived Stem Cells Versus Their Lysate in Ameliorating Erectile Dysfunction in a Rat Model of Type 2 Diabetes

**DOI:** 10.3389/fphys.2022.854949

**Published:** 2022-05-10

**Authors:** Rania A. Galhom, Horeya Erfan Korayem, Mahrous A. Ibrahim, Ahmed Abd-Eltawab Tammam, Mohamed Mansour Khalifa, Eman K. Rashwan, Manal H. Al Badawi

**Affiliations:** ^1^ Human Anatomy and Embryology Department, Faculty of Medicine, Suez Canal University, Ismailia, Egypt; ^2^ Tissue Culture Unit, Centre of Excellence in Molecular and Cellular Medicine (CEMCM), Suez Canal University, Ismailia, Egypt; ^3^ Histology and Cell Biology Department, Faculty of Medicine, Suez Canal University, Ismailia, Egypt; ^4^ Forensic Medicine and Clinical Toxicology, College of Medicine, Jouf University, Sakaka, Saudi Arabia; ^5^ Forensic Medicine and Clinical Toxicology Department, Faculty of Medicine, Suez Canal University, Ismailia, Egypt; ^6^ Medical Physiology Department, College of Medicine, Jouf University, Sakaka, Saudi Arabia; ^7^ Medical Physiology Department, Faculty of Medicine, Beni-Suef University, Beni-Suef, Egypt; ^8^ Department of Human Physiology, Faculty of Medicine, Cairo University, Cairo, Egypt; ^9^ Department of Human Physiology, College of Medicine, King Saud University, Riyadh, Saudi Arabia; ^10^ Medical Physiology Department, Faculty of Medicine, Al-Azhar University, Assiut, Egypt; ^11^ Human Anatomy and Embryology Department, Faculty of Medicine, Helwan University, Cairo, Egypt

**Keywords:** urine-derived stem cells (UDSCs), lysate, erectile dysfunction, diabetes mellitus, rats

## Abstract

**Background:** Diabetic erectile dysfunction (DED) is a significant consequence of diabetes mellitus, and it is a multifactorial phenomenon that has no definitive treatment until now. Many therapeutic options provide symptomatic improvement rather than addressing the underlying etiology or restoring normal function. Stem cell (SC) therapy represents a potential hope in DED management. It is well established that the regenerative effect of stem cells can be attained by their paracrine action and their ability to differentiate into many cell lineages, including endothelial and smooth muscle cells. Hence, we tried to compare the effects of transplantation of urine-derived stem cells (USCs) or their lysate (USC-L) into the corpora cavernosa (CCs) of rats with DED.

**Materials and Methods:** A total of 55 adult male Wistar rats were included in this study. USCs were obtained from ten healthy rats. Another ten rats did not subject to any intervention and served as a control (group I). Type 2 DM and DED were induced in the remaining 35 rats, but DED was tested and proved in only 24 rats, which were randomly divided into three groups (*n* = 8 in each). The DED group (group II) and either USCs (2 × 10^6^ cells) or their lysate (200 μl) were transplanted into the CCs of each rat in the other two groups (groups III and IV), respectively.

**Results:** Although the DED rats exhibited deterioration in all copulatory functions as compared to the control group, our histopathological, immunohistochemical, and morphometric results revealed that both USCs and USC-L have significantly restored the cavernous spaces, the ultrastructures of the endothelium that line the cavernous spaces, collagen/smooth muscle ratio, and the mean area percentage of α-SMA in the CCs as compared to DED rats. A respectable number of USCs was detected in the CCs of group III at the 4th week after transplantation, but this number significantly declined by the 8th week.

**Conclusion:** Both USCs and USC-L can repair the structure and ultrastructure of CCs and improve the copulatory functions in the DED rat model. However, USC-L could be better used in DED to guard against the strange behavior of USCs after transplantation and their decreased survivability with time.

## Introduction

Erectile dysfunction (ED) is a major complication of type 2 diabetes mellitus (DM), which has a robust impact on patients’ quality of life and their families. It was evident that the prevalence of diabetic erectile dysfunction (DED) among the diabetic male patients has reached 78.7% (Sondhi et al., 2018), which is much higher than the overall percentage of men suffering from ED, approximately 15%–20%, among the general male population (Rosen, 2000). Older men with diabetes mellitus more easily exhibit ED, which has a poor response to drugs. Furthermore, there is a significant correlation between the duration of DM illness and the severity of ED (Sondhi et al., 2018). Chen et al. (2017) also reported that diabetic men are thought to develop ED 10–15 years earlier than non-diabetics.

Globally, diabetic persons were assessed to be 9.3% of the whole population (463 million) in 2019, anticipated to be 10.2% (578 million) by 2030 and 10.9% (700 million) by the end of 2045, with type 2 diabetes forms about 90% of all diabetic cases (Saeedi et al., 2019). Hence, DED is thought to be a seriously persistent inability that prevents satisfactory sexual performance and imposes a lot of pressure and burden on society and families worldwide.

The pathophysiology of DED is aptly multifactorial with vascular, neurological, and hormonal aspects. Among these factors, endothelial dysfunction is a well-known pillar in the pathophysiology of DED. Many therapeutic strategies for ED include oral medications, intracavernosal injections, vacuum erection tools (penile pump), and semi-rigid or inflatable penile implants. However, all those therapeutic options afford only symptomatic improvement without repairing the pathogenesis or re-establishing the standard penile structure and function (Matz et al., 2019). Despite that phosphodiesterase type-5 inhibitor (PDE5i) is the most widely used and the first-line medication in the management of ED, it showed diminished therapeutic efficacy in the treatment of DED. This is probably caused by the decline in nitric oxide (NO) production and its concentration within the endothelial cells hence affecting their functions (Chung, 2019). Therefore, new therapeutic strategies that prompt restoring of the endothelial functions are critically required.

Stem cell (SC) therapy represents a promising new hope for DED treatment. Hence, many researchers identified the therapeutic effects of numerous types of stem cells, including mesenchymal stem cells (MSCs), such as rat bone marrow-derived mesenchymal stem cells (BM-MSCs), vascular endothelial growth factor (VEGF), transfected BM-MSCs, and urine-derived stem cells (USCs) (Matz et al., 2019). USCs can be isolated from human urine aliquots using a low-cost, simple isolation method, not requiring complex elements or invasive procedures of collection in comparison to the other sources of stem cells. Moreover, USCs are a superior choice as cell sources because they have favorable genetic information, native property of progenitor cells, and inherent multipotent potentiality. USCs also exhibit many characteristics of MSCs, and they can differentiate into many cell lineages, including endothelial and smooth muscle cells (SMCs) (Nimshitha et al., 2018).

Furthermore, the injection of SCs into the corpora cavernosa to treat ED appears to be a forthright and reasonable preference due to their anticipated regenerative impact attained either by their paracrine effect through the local secretion of growth factors or *via* the migration to the major pelvic ganglia to enhance the proliferation and differentiation of the inhabitant types of progenitor cells. They also promote the recovery of injured, fibrosed, or lost tissue *via* the production of anti-apoptotic, proangiogenic, and neurotrophic factors, rather than their trans-differentiation into various cell types (Ouyang et al., 2019). The first reported clinical trial of SC therapy in DED showed a significant increase in penile rigidity after a single trans-penile injection of umbilical cord blood SCs. Penile rigidity was retained for more than 6 months in those patients. However, the erection was not hard enough for sexual penetration, indicating that the amount and the single administration of SCs were probably insufficient for adequate penile rigidity and to restore the penile copulatory functions (Al Demour et al., 2018). The same results were reported when bone marrow mononuclear cells were used to treat some ED cases following radical prostatectomy (Damkier et al., 2016). Although prior studies proved that SC therapy is a valuable option in DED management, the optimal strategy has not been determined yet. Therefore, new ideas for DED treatment are needed to design the most efficient approach for future experimental research (Matz et al., 2019).

In this study, we simply try to investigate the underlying mechanisms of using stem cells in the treatment of DED either by their multipotentiality and homing capacity or their paracrine effects by comparing the effectiveness of urine-derived stem cells and their lysate in improving erectile dysfunction in the type 2 diabetic rat model where the research work is still insufficient.

## Material and Methods

### Animals

The experiments were performed on 55 young adult male Wistar rats weighing 200–250 g. The rats were examined for health status and acclimated to the standard laboratory conditions (25 ± 2°C, 12-h light–dark cycle, and humidity of 40% ± 10%) with free access to standard rodent laboratory chow and water for 2 weeks before the start of the study. All experimental procedures and animal maintenance were conducted following the accepted standards of animal care of the medical research center, Ain Shams University. The laboratory work was performed in the tissue culture laboratory of Pancreatic Cell Culture and Diabetic Research center, Ain Shams University Hospitals.

### Study Design

A total of 10 healthy adult male Wistar rats were used as donors for USCs. Another 10 rats were considered the control group (group I) and were not subject to any intervention until the end of the study. The remaining 35 rats were subjected to the induction of type 2 DM by a high-fat diet (HFD), followed by an intraperitoneal injection of streptozotocin (STZ); diabetes was established in 31 out of them and was confirmed by the fasting blood glucose and insulin tolerance test. DED was tested and proved in only 24 out of the diabetic rats, further randomly divided into three groups (*n* = 8 in each). Diabetic-induced erectile dysfunction group “DED” (group II): each rat was injected intracavernously with 0.2 ml phosphate-buffered saline (PBS). Diabetic-induced erectile dysfunction/USC-treated group (group III): each rat was subjected to the intra-cavernous injection of USCs (2 × 10^6^ cells) suspended in 0.2 ml PBS. Diabetic-induced erectile dysfunction/USC-L-treated group (group IV): each rat was subjected to the intra-cavernous injection of USC-L (200 μl of lysate). After 4 and 8 weeks of injection, erectile functions and sexual behavior were assessed, and the animals were euthanized by the end of the 8th week after transplantation. The penile tissue was harvested from all animals for further histopathological, immunohistochemical, and morphometric studies. The following illustration shows the steps and the duration of each phase in the study.

### Induction of Type 2 DM in a Rat Model and Confirmation of Diabetic Erectile Dysfunction

A total of 35 rats were fed HFD 4 weeks before the induction of type 2 diabetes. The diet was purchased from the medical research center, Ain Shams University. Its composition was 24% fat, 41% carbohydrates, and 24% protein with a total caloric value of 4.7 Kcal/g. Two doses of 30 mg STZ/kg/BW, dissolved in normal saline (Sigma Chemical Co., St. Louis, MO) with 7 days of interval, were intraperitoneally injected to induce type 2 diabetes. An animal was considered diabetic when its blood glucose was >280 mg/dl for 2 consecutive days (Chen et al., 2017). Body weight was assessed every week starting from the beginning of HFD until the end of the study. An insulin tolerance test was performed after 8 weeks of the induction of diabetes to test the insulin resistance of the animal. According to (Chen et al., 2017), 1 IU/ml of bovine insulin was intraperitoneally injected into each rat, followed by measuring blood glucose at 0, 15, 30, 60, 90, and 120 min after insulin injection to attain the blood glucose response curve. The percentage of glucose level was calculated in relation to the baseline measures before insulin injection. At the same time, orbital blood samples from all rats were obtained to assess fasting blood glucose, total cholesterol, and triglyceride. The incidence of ED was confirmed following the method of Brien et al. (2000) by “apomorphine screening test” using a mixture of apomorphine hydrochloride (APO) (Sigma Chemical Co., St. Louis, MO); 80 μg/kg dissolved in a vehicle of 100 µg ascorbic acid in normal saline 0.9% (l ml/kg). This compound was subcutaneously injected into the back of the neck, and then erectile and yawning responses to APO were recorded for 30 min. This test was performed on the animals 4 weeks after the induction of diabetes (from the first dose of STZ) and repeated on all animals 8 weeks after the transplantation of USCs and USC-L.

### Urine Collection From the Donor Rats

The urine was collected from the donor rats after strict disinfection of the skin of the lower abdomen and the external genitalia. Urination was induced manually by applying a gentle trans-abdominal pressure over the bladder to overcome the normal urethral pressure. The first few urine drops from each rat were discarded, while the remaining urine sample was forced to come out into a sterile disposable plastic petri dish and then aspirated and poured off into a Falcon tube (Kurien et al., 2004). The urine was collected from each rat of the donor animals three times/day. The obtained urine was immediately taken and processed in the tissue culture laboratory each time. The collected urine volume from donor animals was 30–100 ml per day.

### Isolation, Culture, and Passaging of Urine-Derived Stem Cells

The collected urine samples were centrifuged (1800 rpm for 10 min), and the resulting cell pellets were washed with PBS (Lonza, Belgium) and supplemented with 1% penicillin/streptomycin (PS) (1:1) (Lonza, Belgium). Then, the cells were plated in 6-multi-well tissue culture plates at about 2000 cells per well with complete medium (CM) composed of 89% DMEM (Lonza, Belgium), 10% fetal bovine serum (FBS) (Seralab, Brazil), and 1% PS. Cell monitoring and follow-up were carried out daily to the primary culture cells (P0). The first medium exchange was after 48 h of seeding, and the subsequent exchanges were performed every 3 days. When reaching confluency (80%–90%), the culture was trypsinized by 0.25% trypsin/EDTA (Gibco, Grand Island, NY) and expanded through three passages (P1–P3). The cells of P3 were used for characterization and transplantation (Ouyang et al., 2014).

### Preparation of USC-L

The modified technique of (Albersen et al., 2010) was used for this purpose; after trypsinization and cell counting, every 2 × 10^6^ cells of P3 were transferred in a separate Falcon tube and centrifuged, and the pellet was obtained. The supernatant medium was discarded from each tube, and the cells were resuspended into 10 ml of deionized H_2_O for half an hour at room temperature to cause cell membrane rupture. Then, three freeze–thaw cycles were performed to obtain cell-free lysate, followed by centrifugation at 2000 rpm for 10 min to eliminate any insoluble fragments. A measure of 200 μl of the lysate was obtained from each tube and frozen at −80°C in a separate Eppendorf tube for each animal until the time of injection.

### Characterization of USCs

The flow cytometry analysis of the isolated USCs of P3 was carried out to show the incidence of expression of the following cell markers: CD73, CD44, and CD45 in the flow cytometry unit, Poisoning Treatment Center, Ain Shams Hospital.

### Intra-Cavernous Injection of USCs and USC-L

After sterilization and anesthesia by sodium pentobarbital (30 mg/kg, IP), the proximal shaft of the penis was tied gently by an elastic rubber band, and 0.2 ml of PBS was injected slowly into the middle of the corpus cavernosum (CC) of each rat in group II. Regarding group III, 2 × 10^6^ USCs labeled with a PKH-26 fluorescent cell linker (Sigma, St. Louis, MO, United States) were suspended with the same amount of PBS and injected into the CC of each rat in this group. Lastly, 200 μl of USC-L was injected into the CC of each animal in group IV. The needle and the rubber band were left in place after injection to prevent leakage (Woo et al., 2011; Ouyang et al., 2014).

### Sample Collection, Histopathological, Immunohistochemical, and Ultrastructure Examination

The animals of all groups were killed 8 weeks after transplantation of USCs and USC-L. The intermediate shafts of the penis of all animals were collected, and half of them were fixed in 10% neutral buffer formalin and embedded in paraffin blocks. The transverse sections of 5 μm thickness were cut and stained with hematoxylin and eosin (H&E) and Masson trichrome stain to evaluate the histopathological changes. These paraffin-embedded sections were further immunohistochemically stained using mouse monoclonal antibodies against alpha-smooth muscle actin (α-SMA) (1:200, Santa Cruz Biotechnology Inc., United States) and desmin (LSAB-plus kit, Dakocytomation, Glostrup, Denmark) following manufacturer’s instructions, after which they were counterstained with Mayer’s hematoxylin. Then, these sections were examined and photographed under a light microscope. The other half of the specimens were carefully cut into small pieces immediately after dissection and fixed in buffered 2.5% glutaraldehyde for 2 h, postfixed in 1% osmic tetroxide, dehydrated in ascending grades of alcohol, and embedded in epoxy resin. Then 1-μm-thick semithin sections were obtained, stained with toluidine blue, examined, and photographed under a light microscope. The ultrathin sections were cut using an ultratome (Reichert Ultracut, Ziess, Germany), stained with uranyl acetate and lead citrate, and examined and photographed under a transmission electron microscope (1230 EXII; JOEL, Tokyo, Japan) (Mycotic Center, Al-Azhar university) (Galhom et al., 2018).

### Morphometric Assessment

ImageJ software (version 1.33–1.34; National Institutes of Health, Bethesda, MD, United States) was used to assess the following parameters (Chen et al., 2017):

1) Field integrated density (IntDen) in Masson trichrome-stained sections. It was determined after subtracting the background noise. The ratio between collagen fibers and smooth muscles (collagen/smooth muscle ratio) was assessed. There were ten different fields from six non-overlapping sections from each sample that were used to calculate this ratio.
2) The average percentage of smooth muscle cells that showed alpha-smooth muscle actin (α-SMA) (brownish immunoreactivity compared to the total tissue area) and the average percentage of the regenerated smooth muscle cells showed that showed immune-positive reactivity to desmin compared to the total tissue area. For each marker, ten different fields from six non-overlapping sections from each sample were also used for this purpose.

### Detection of USCs in the Corpus Cavernosum

Before USCs transplantation, the cells were incubated with the immunofluorescence dye, PKH-26, in accordance with the manufacturer’s instructions. PHK-26-labeled USCs were identified by immunofluorescence microscopy in the corpus cavernosum of group III rats 4 weeks and 8 weeks after transplantation (Woo et al., 2011).

### Sexual Behavior (Mating Behavior and Copulatory Functions)

In a cage measured 60 × 40 × 30 cm, with a glass side, and illuminated with a faint light, the male rat was placed into the cage an hour before the test, then the female was introduced inside (induction of the behavioral estrus in the female rat took place by intraperitoneal injection of 20 μg/kg estradiol benzoate 24 h before the test). Both were observed for half an hour, and the following copulatory parameters were measured:

Mount latency (ML): The time from the introduction of the female rat into the cage until the first mount of the male rat (measured in seconds).

Intromission latency (IL): The time from the introduction of the female rat until the ﬁrst intromission by the male rat (measured in seconds).

Mount frequency (MF): The number of mounts before ejaculation.

Intromission frequency (IF): The number of intromissions before ejaculation.

Ejaculation latency (EL): The time from the ﬁrst intromission until the first ejaculation (measured in seconds).

The total number of ejaculation (TE): The total number of ejaculations occurring during the test duration (30 m).

Post-ejaculatory interval (PEI): The time from the ﬁrst ejaculation until the next intromission by the male rat (measured in seconds).

This test was performed twice, at the beginning of the study to exclude the sexually inactive male rats, then at the 8th week after transplantation of USCs and USC-L to all rats in all groups. When the animal did not mount during the first 10 min of the test, it was excluded, and the test was considered negative (Scarano et al., 2006a; Nathani et al., 2019).

### Statistical Analysis

Data were analyzed using Statistical Package for the Social Science (SPSS) 18.0 statistical software (SPSS Inc., Chicago, IL, United States). Numeric data with normal distribution were presented as mean ± standard error of mean (SEM). Means were compared using the one-way ANOVA test. The significance between the study groups was tested using the Tukey–Kramer post hoc test. Differences among groups were considered significant when *p*-value < 0.05. The markers’ expression was presented as box plots (R Studio version 1.0.136 – © 2009–2016, Inc.).

## Results

### General Characteristics

**FIGURE 1:**
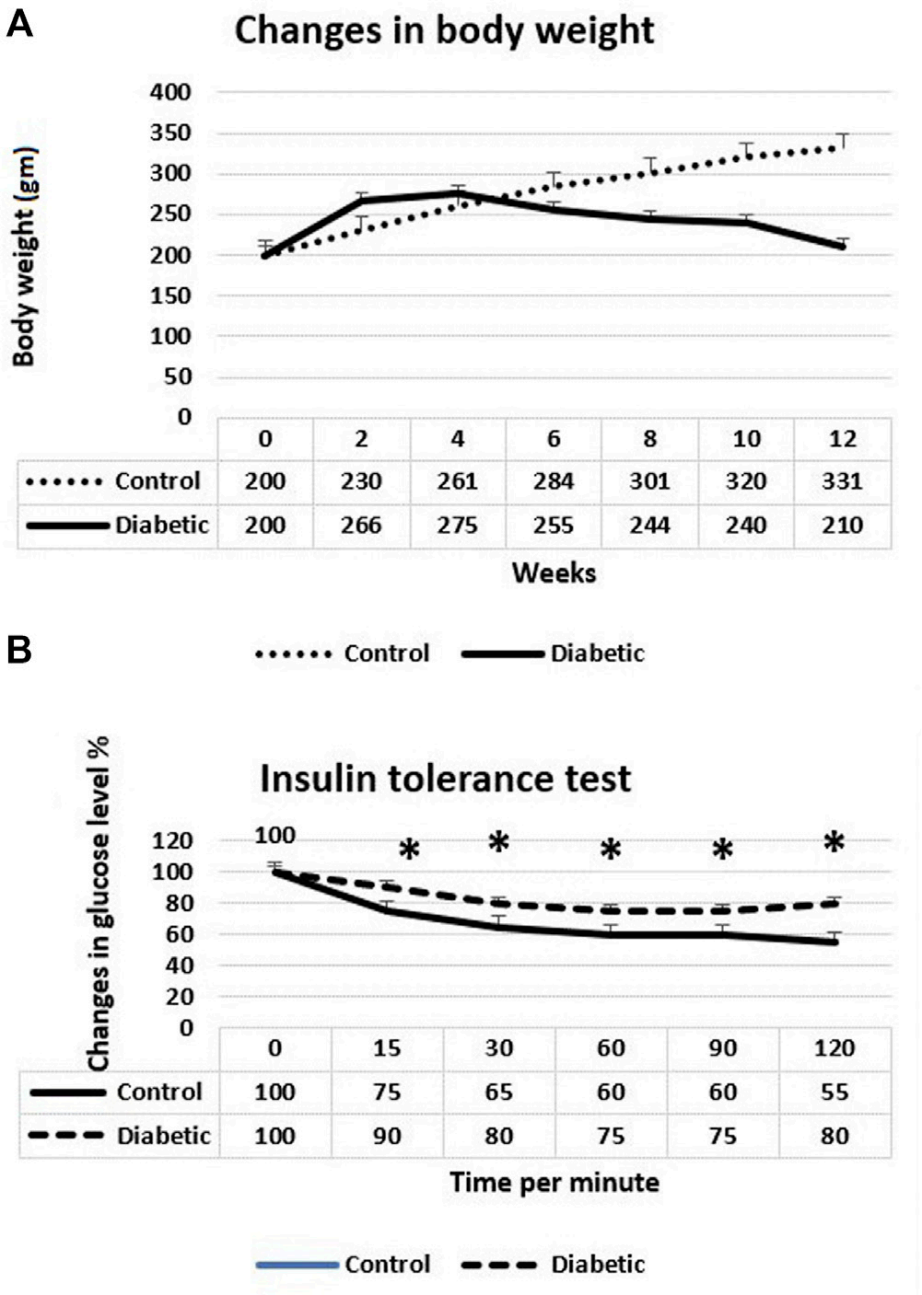
**(A)** Changes in body weights of control and diabetic rats. The diabetic rats showed a significant decrease of their weight 8, 10, and 12 weeks after the induction of diabetes. **(B)** Insulin tolerance test at the 8th week after the induction of diabetes. The diabetic rats showed a significant weak response to insulin. *p < 0.05.

**FIGURE 2:**
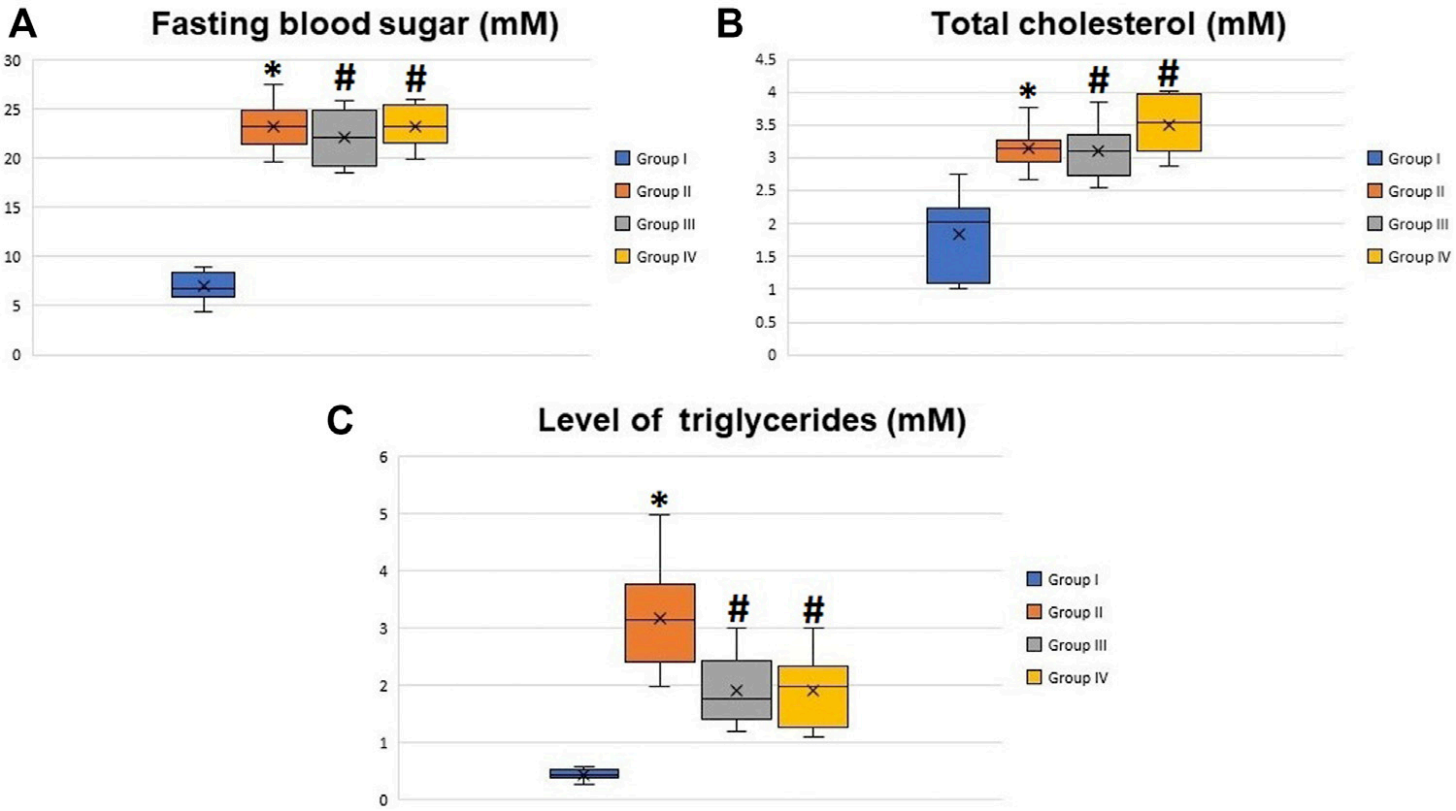
Boxplot graphs showing the mean values of fasting blood glucose **(A)**, total cholesterol **(B)**, and triglyceride levels **(C)** in all groups 8 weeks after the induction of diabetes. All the measured values showed a significant increase in diabetic rats (groups II, III, and IV) compared to the control group (group I) and non-significant difference between groups II, III, and IV. *Significant difference compared to group I (*p* < 0.05). #Significant difference compared to group II (*p* < 0.05).

Body weights of the animals and insulin tolerance test are shown in Figures 1A,B, respectively. The preliminary mean body weights of the rats in the control (group I) and diabetic groups (groups II, III, and IV) were the same. Still, the diabetic rats exhibited a significant decrease in body weight compared to the controls at the 8th, 10th, and 12th weeks after the induction of diabetes (the start point is the first dose of STZ) (*p* < 0.05). Regarding the insulin tolerance test, blood glucose levels significantly declined in normal rats at 15, 30, 45, 60, 90, and 120 min after intraperitoneal injection with insulin. In contrast, the response to insulin in diabetic rats was significantly decreased. The fasting blood glucose levels in the diabetic rats at the 8th week after the induction of diabetes were about four times higher than the glucose levels in the control rats (*p* < 0.001), with non-significant differences between the diabetic groups (groups II, III, and IV) (Figure 2A). The serum levels of total cholesterol and triglycerides revealed a significant increase in the diabetic rats (groups II, III, and IV) in comparison to the control group (group I) (*p* < 0.05 and *p* < 0.01), respectively (Figures 2B,C).

### Isolated USC Morphology and Phenotypic Characterization

**FIGURE 3:**
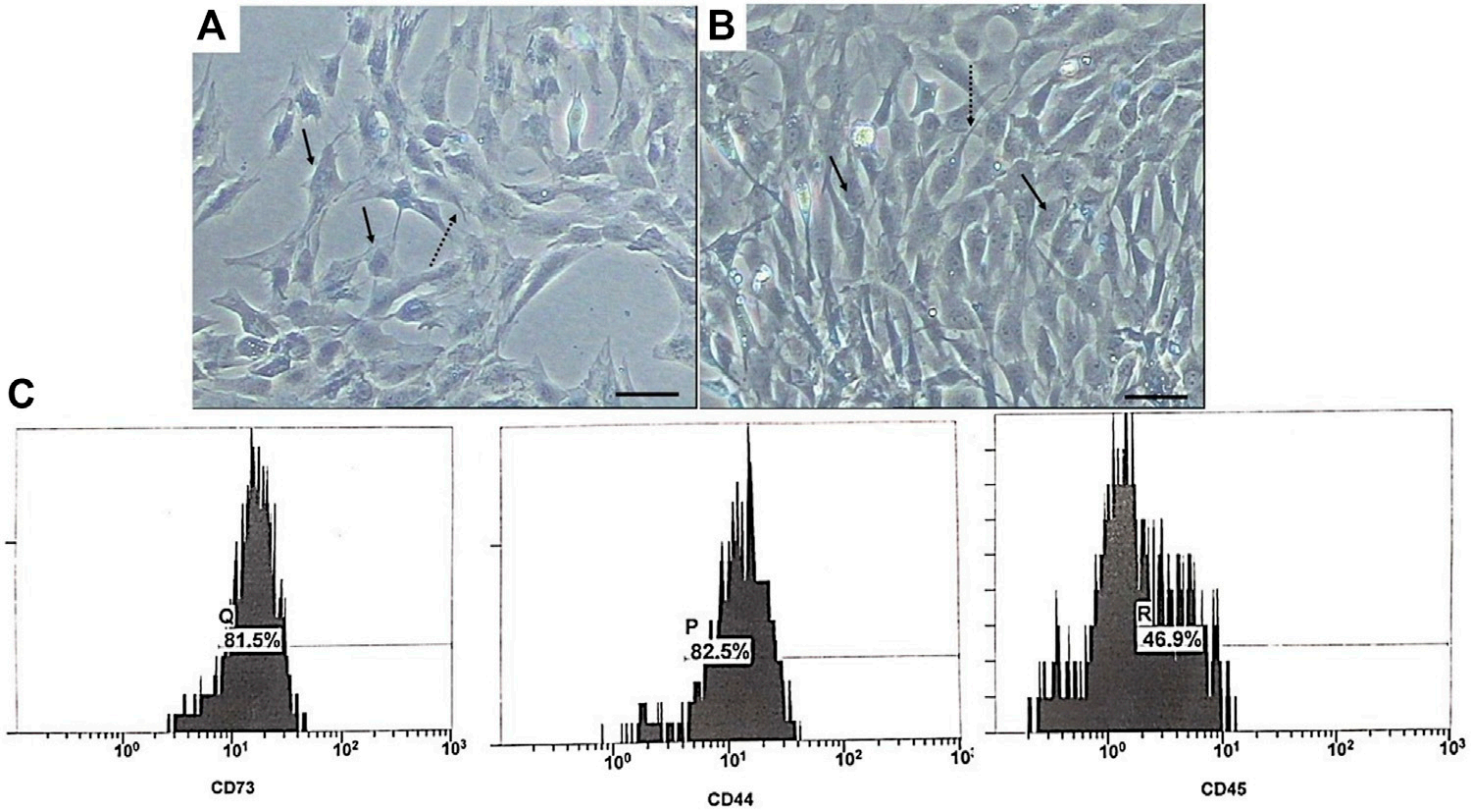
Phase contrast photomicrographs of USCs during P1 **(A)** and P3 **(B)**. The cells exhibited fibroblast-like appearance with short cytoplasmic processes (dashed arrow), and their nuclei were vesicular with multi nucleoli (arrow). Scale bar: 100 μm. **(C)** Flow cytometry phenotypic analysis of USCs.

The isolated USCs showed a plastic adherent property. A few numbers of them were noticed attached to the floor of the tissue culture flask 4 days post-culture. They took about 15 and 20 days to reach 50 and 90% confluency, respectively. Most of the cells showed short cytoplasmic processes, vesicular nucleus, and multiple nucleoli during the first passage (P1) (Figure 3A) and acquired a rice-shaped appearance during the third passage (P3) (Figure 3B). Most of the isolated cells expressed the mesenchymal stem cell markers CD 73 (81.55%) and CD 44 (82.5%). Only 46.9% of them expressed the hematopoietic stem cell marker CD 45 (Figure 3C).

### Histopathological Results

**FIGURE 4:**
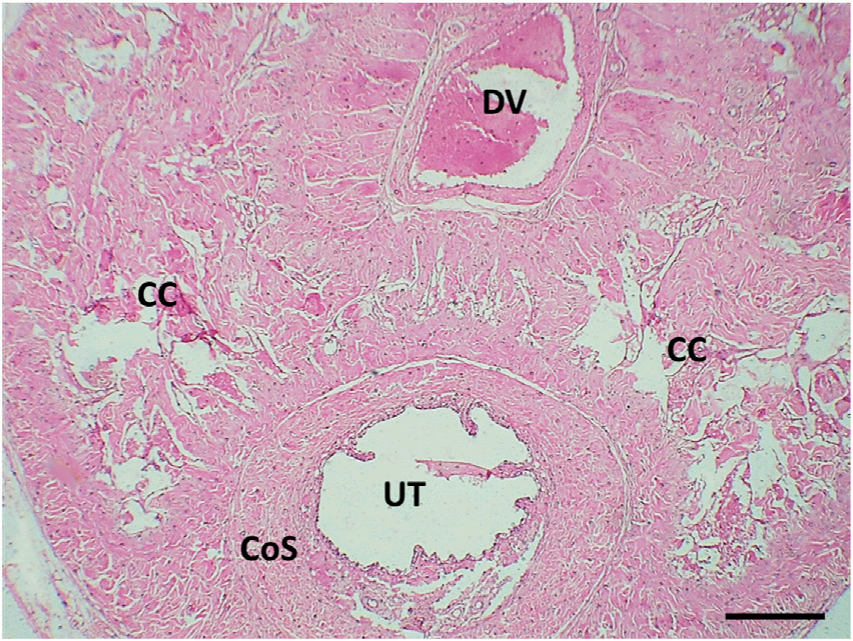
Histological transverse section of the penis of the control rat showing the 2 corpora cavernosa (CC) located dorsal to the corpus spongiosum (CS). UT: urethra. DV: deep dorsal vein of the penis. H&E X40. Scale bar: 500 μm.

**FIGURE 5:**
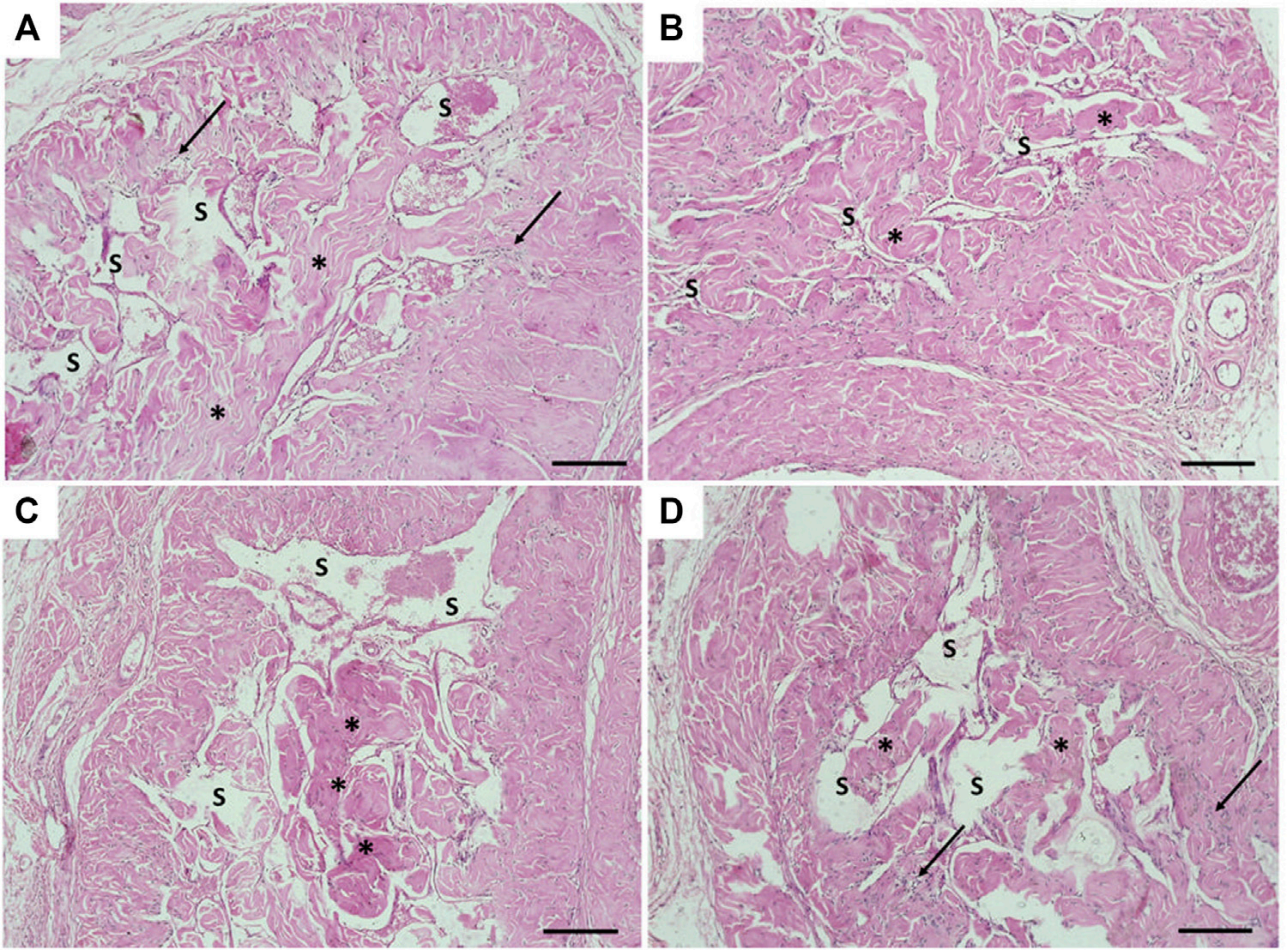
Histological transverse sections of the penises of all rats in all groups. **(A)** Corpus cavernosum in the control group (group I) appeared with wide cavernous spaces and formed by smooth muscles and collagen fibers. **(B)** Narrowing of the cavernous spaces the in diabetic group (group II). **(C)** and **(D)** showing almost restoration of cavernous spaces in groups III and IV, respectively. CS: cavernous spaces. Arrow: Smooth muscles. Asterisk: collagen fibers. H&E X100. Scale bar: 100 μm.

The transverse section in the mid-shaft of the rat penis showed three cavernous cylindrical bodies (corpora), a pair of corpora cavernosa (CCs), and the corpus spongiosum (CoS) that surrounds the urethra (Figure 4). The corpora cavernosa of the control rats contained wide irregular vascular cavernous spaces (CS); composed of collagen fibers intertwined with smooth muscle and lined by endothelium (Figure 5A). The CCs of the diabetic rats showed marked narrowing of the CS, which appeared to be invaded by the bands of thick collagen fibers (Figure 5B). The rats of groups III and IV (Figures 5C,D), respectively, exhibited apparent CS restoration regarding their size and structure. However, a few thick collagen fibers are still being seen obliterating them in group III rats (Figure 5C).

### Detection of Collagen/Smooth Muscle Ratio

**FIGURE 6:**
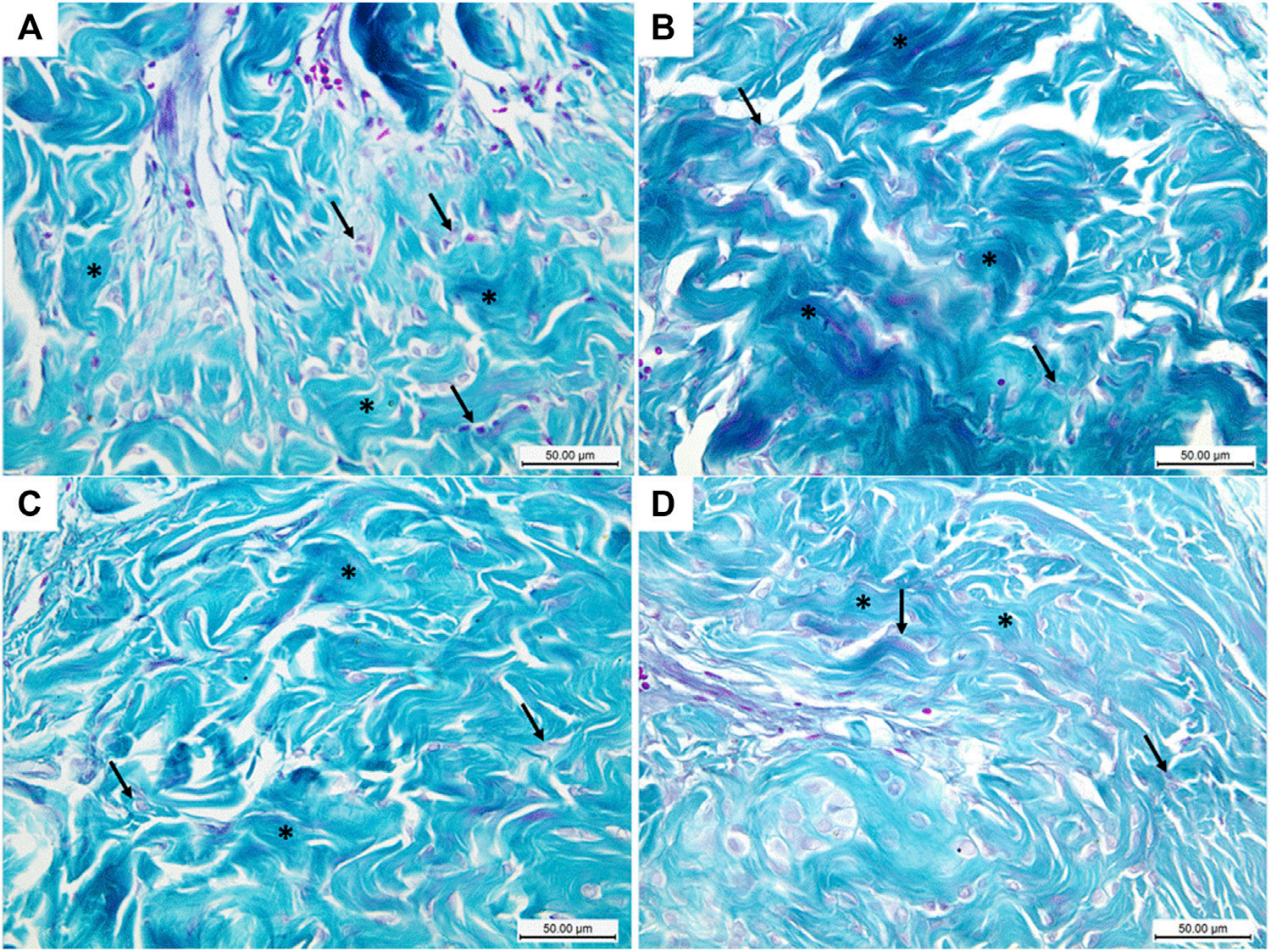
Histological transverse sections of the penises of all rats in all groups stained with Masson trichrome stain; blue and the red colors indicate collagen and smooth muscles, respectively. The diabetic rats showed increase collagen expression **(B)** in comparison to the control rats **(A)**. Collagen expression in groups III and IV **(C)** and **(D)**, respectively, greatly resembles its expression in the control group **(A)**. Masson trichrome X400. Scale bar: 50 μm.

**FIGURE 7:**
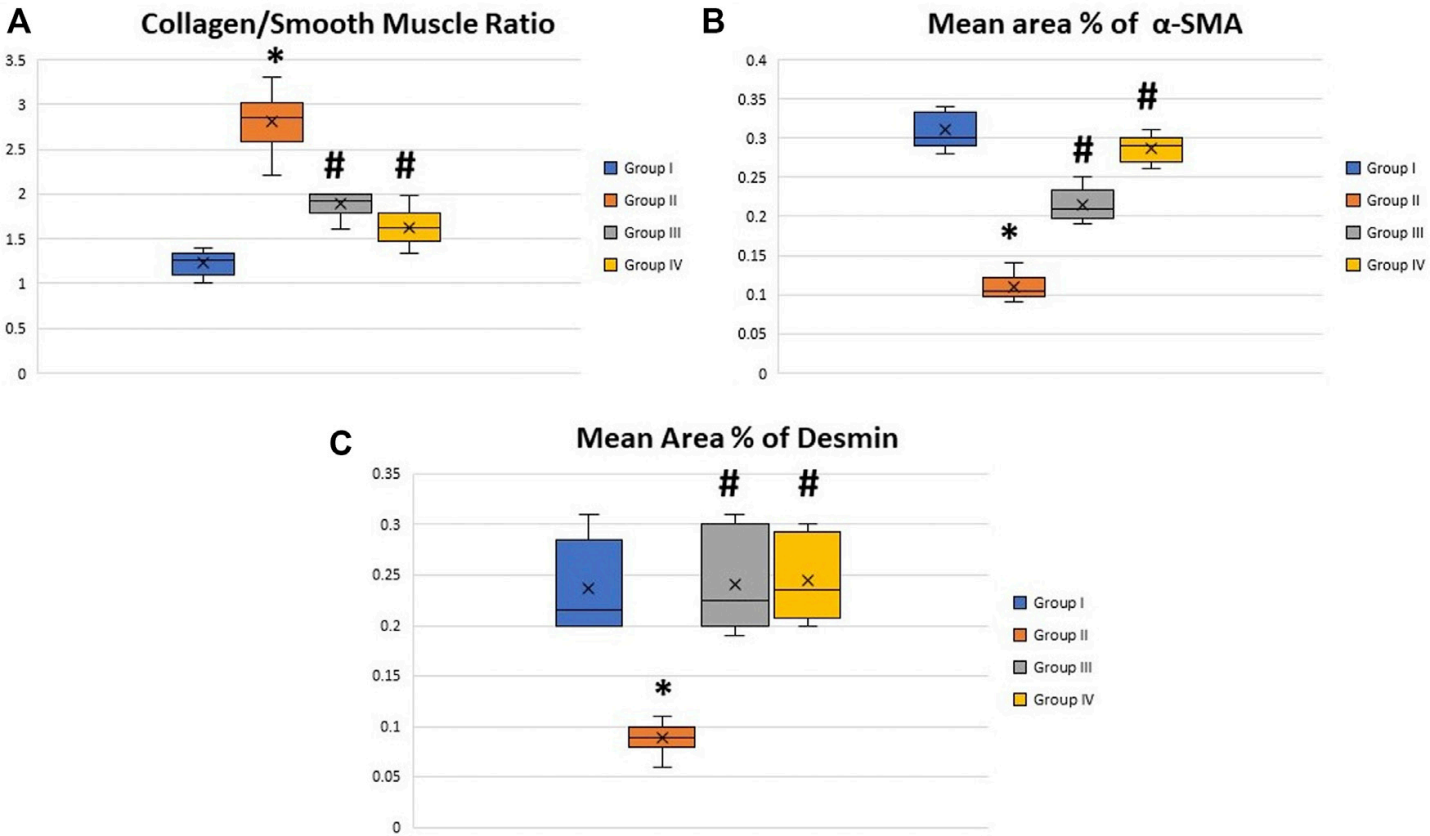
Boxplot charts showing the collagen/smooth muscle ratio **(A)**, the mean area percentage of α-SMA **(B)**, and the mean area percentage of desmin **(C)** in the corpora cavernosa of all rats in all groups. The expression of α-SMA and desmin significantly decreased in the diabetic rats (group II), while the collage/smooth muscle ratio significantly increased in the same group. Both treated groups (groups III and IV) showed almost restoration of the control values. *Significant difference compared to group I (*p* < 0.05). #Significant difference compared to group II (*p* < 0.05).

Masson trichrome-stained histological sections of the CC of diabetic rats (group II) showed a large amount of collagen fibers deposition and penile fibrosis (Figure 6B) compared to the sections of the control group (group I) (Figure 6A), while the corpora cavernosa of both treated groups (groups III and IV) (Figures 6C,D, respectively) exhibited almost similar appearance to the control group (group I) regarding their collagen fiber content. The collagen/smooth muscle ratio increased significantly in the diabetic rats (group II) (2.8 ± 0.4) (*p* < 0.05) in comparison to the control group (group I) (1.3 ± 0.2). The same ratio decreased significantly in groups III and IV (1.9 ± 0.3 and 1.7 ± 0.4), respectively (*p* < 0.05), in comparison to the diabetic group without a significant difference between groups III and IV (*p* < 0.1) (Figure 7A).

### Assessment of α-SMA and Desmin Expression in CC

**FIGURE 8:**
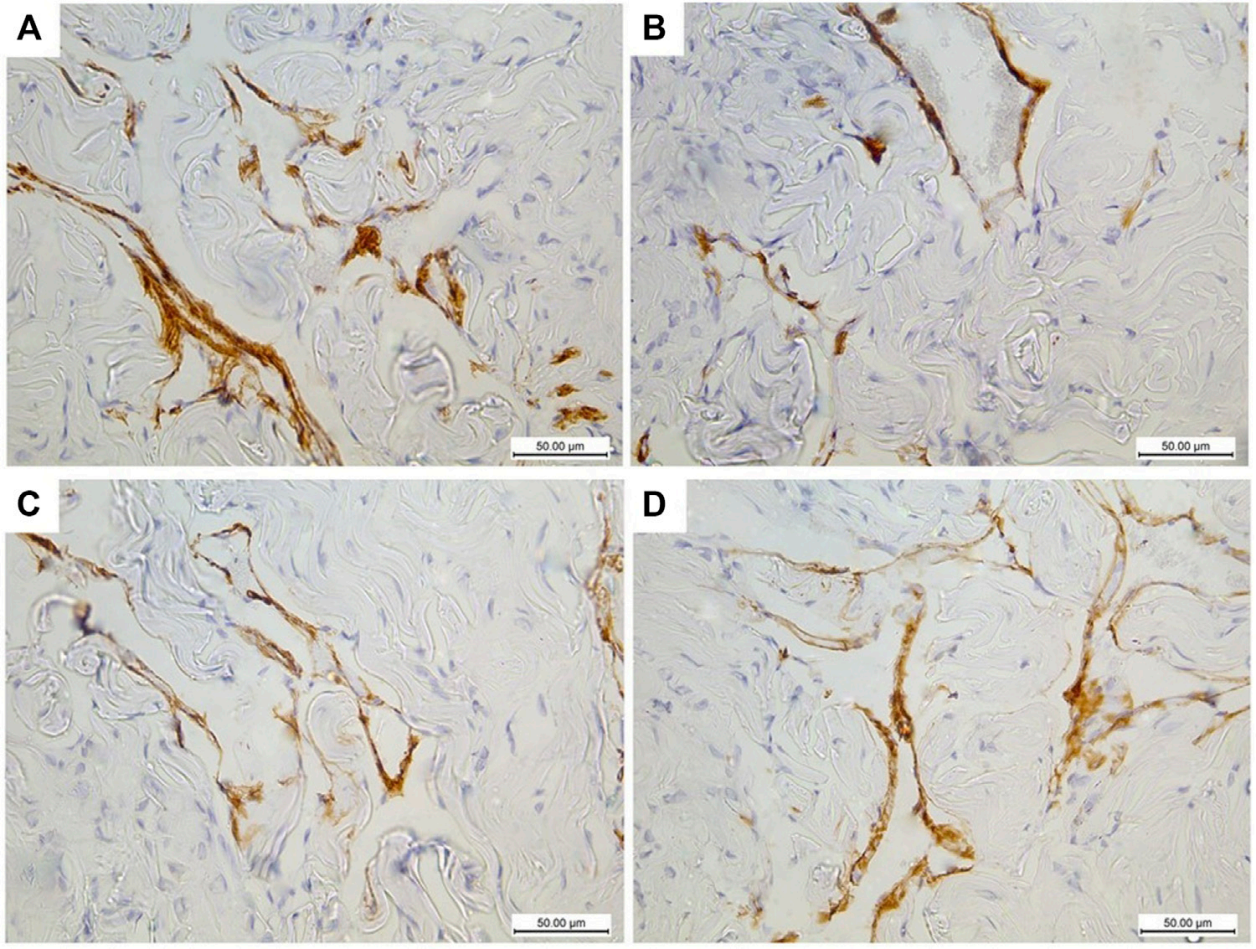
Histological transverse sections of the penises of all rats in all groups immunohistochemically stained with anti-α-SMA antibody showing diminution of its expression in diabetic group (group II) **(B)** in comparison to the control group (group I) **(A)** and restoration of its expression in groups III and IV **(C)** and **(D)**, respectively. X400. Scale bar: 50 μm.

**FIGURE 9:**
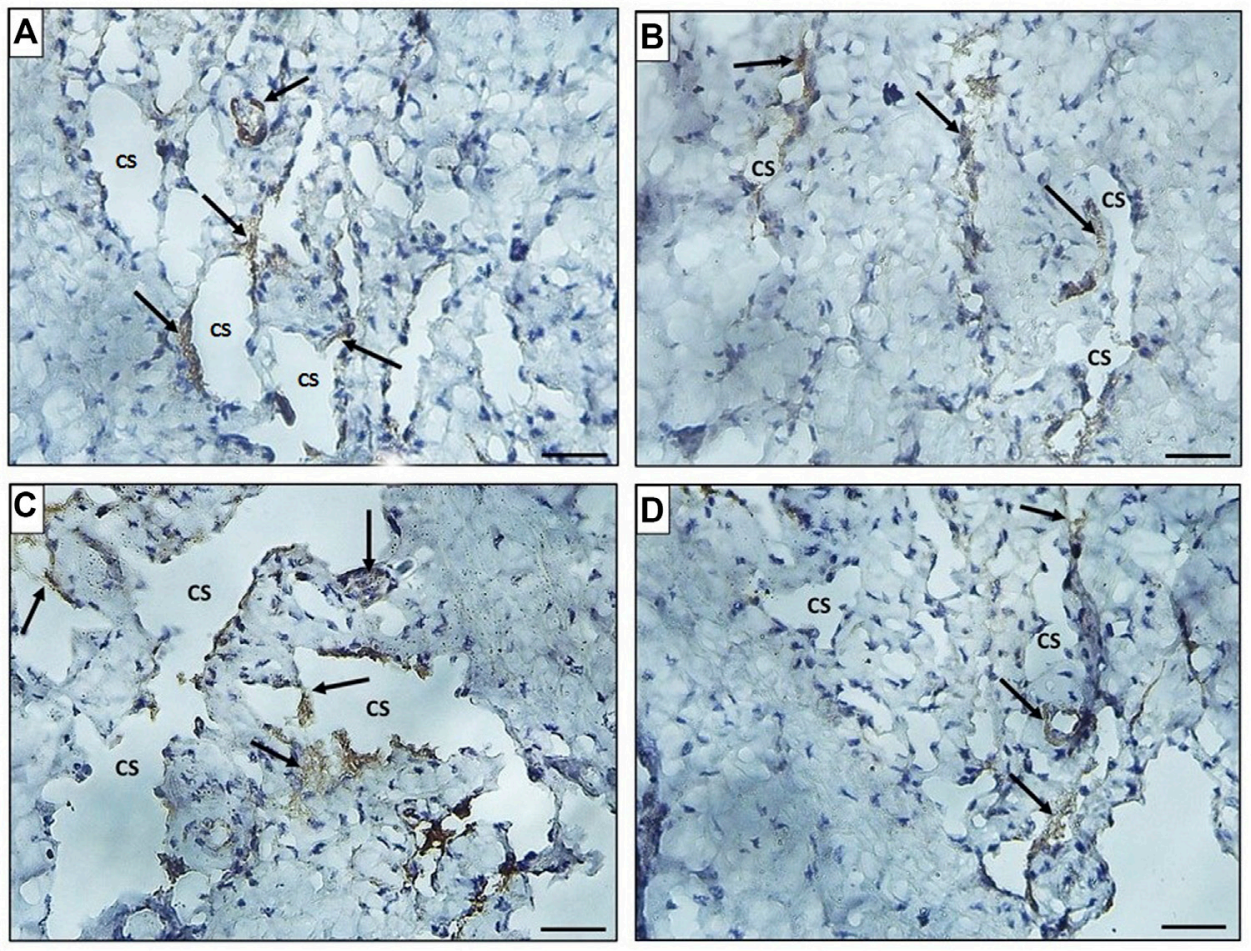
Histological transverse sections of the penises of all rats in all groups immunohistochemically stained with anti-desmin antibody showing immunopositive cytoplasmic and nuclear reaction in smooth muscle cells around the cavernous spaces. The expression decreased in diabetic group (group II) **(B)** in comparison to the control group (group I) **(A)** and restoration of its expression was noticed in groups III and IV **(C)** and **(D)**, respectively. CS: cavernous spaces. Arrow: positively stained cells around the CS. X400. Scale bar: 50 μm.

The immunohistochemically stained sections of the penises of diabetic rats (group II) revealed marked decreased immunopositivity for α-SMA in the CC (Figure 8B) compared to the control group (group I) (Figure 8A). In contrast, the treated groups (groups III and IV) exhibited a relatively similar appearance to the control group regarding this marker expression (Figures 8C,D), respectively. The quantification of these data showed a significant decrease in the mean area percentage of α-SMA in the immunohistochemical-stained sections of diabetic rats (group II) (0.1 ± 0.002) (*p* < 0.05) compared to the control group (group I) (0.3 ± 0.001). This level increased significantly in groups III and IV (0.23 ± 0.6 and 0.28 ± 0.4), respectively (*p* < 0.05), in comparison to the diabetic group with no significant difference between groups III and IV (*p* < 0.1) (Figure 7B). Desmin also was expressed primarily in the muscular tissues of the CC of the control group (group I), mainly in the cytoplasm and partially in the nuclei of cells around the cavernous spaces (Figure 9A). The least immunopositivity was recognized in the diabetic group (group II) (Figure 9B). In comparison, the sections of groups III and IV were almost like those of the control group (Figures 9C,D), respectively. The morphometric assessment of the desmin-stained sections showed a significant decrease in its expression in group II (0.08 ± 0.01) compared to the control group (0.23 ± 0.04) (*p* < 0.05). On the other hand, the area percentage of the same marker showed a significant increase in groups III and IV (0.24 ± 0.04 and 0.24 ± 0.03), respectively (*p* < 0.05), with no significant difference between them (*p* < 0.1) (Figure 7C).

### Transmission Electron Microscopic Results

**FIGURE 10:**
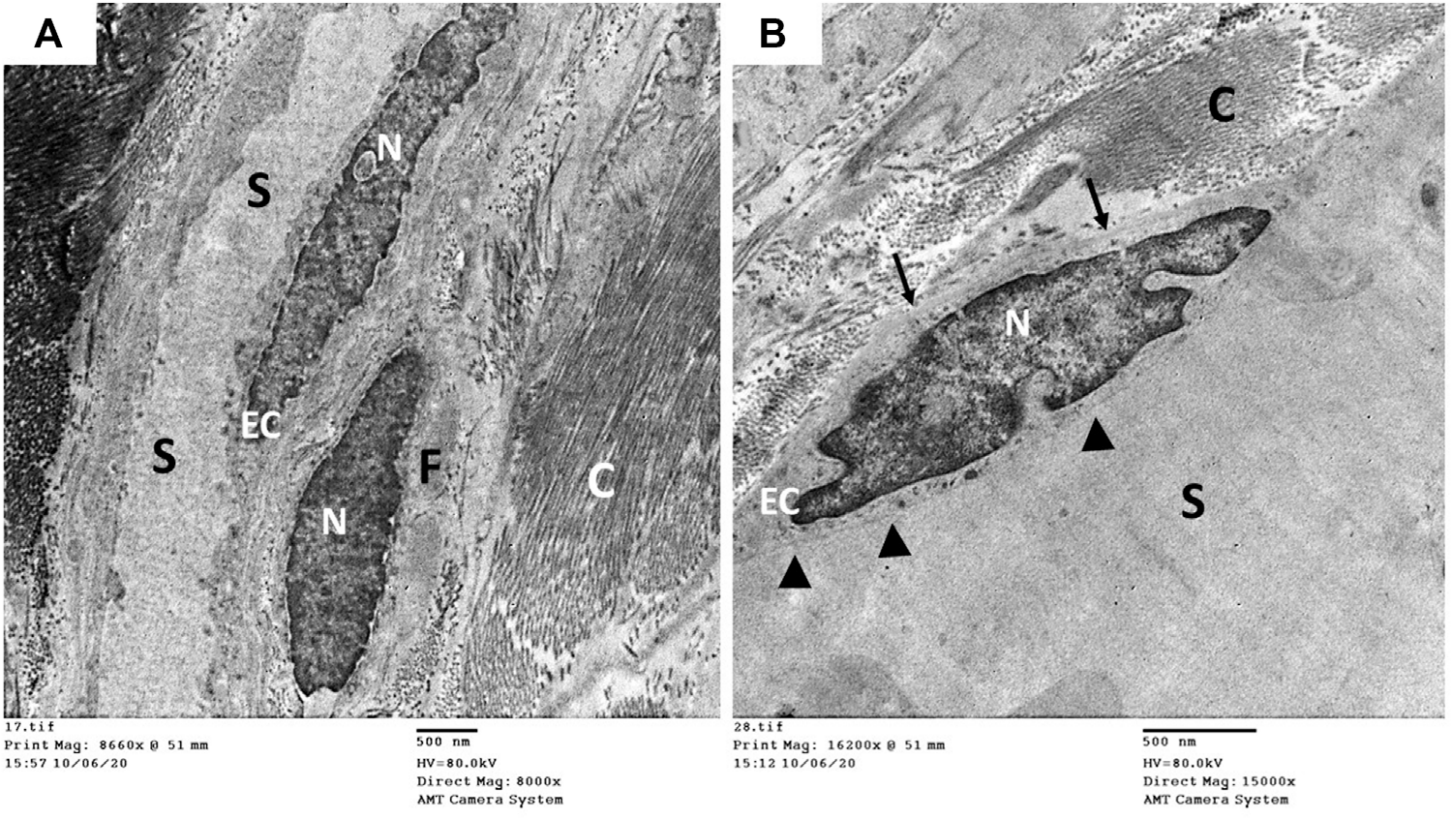
Transmission electron microscopic pictures of the corpus cavernosum of control rat showing **(A)** wide cavernous spaces (S) lined with endothelial cells (EC) and surrounded by mature collagen fibers (C) and fibroblasts (F). **(B)** The lining endothelium showing well identified nucleus (N), narrow basal lamina (arrow), and smooth cavernous surface (arrowhead) but lacking the pinocytic vesicles in the cytoplasm.

**FIGURE 11:**
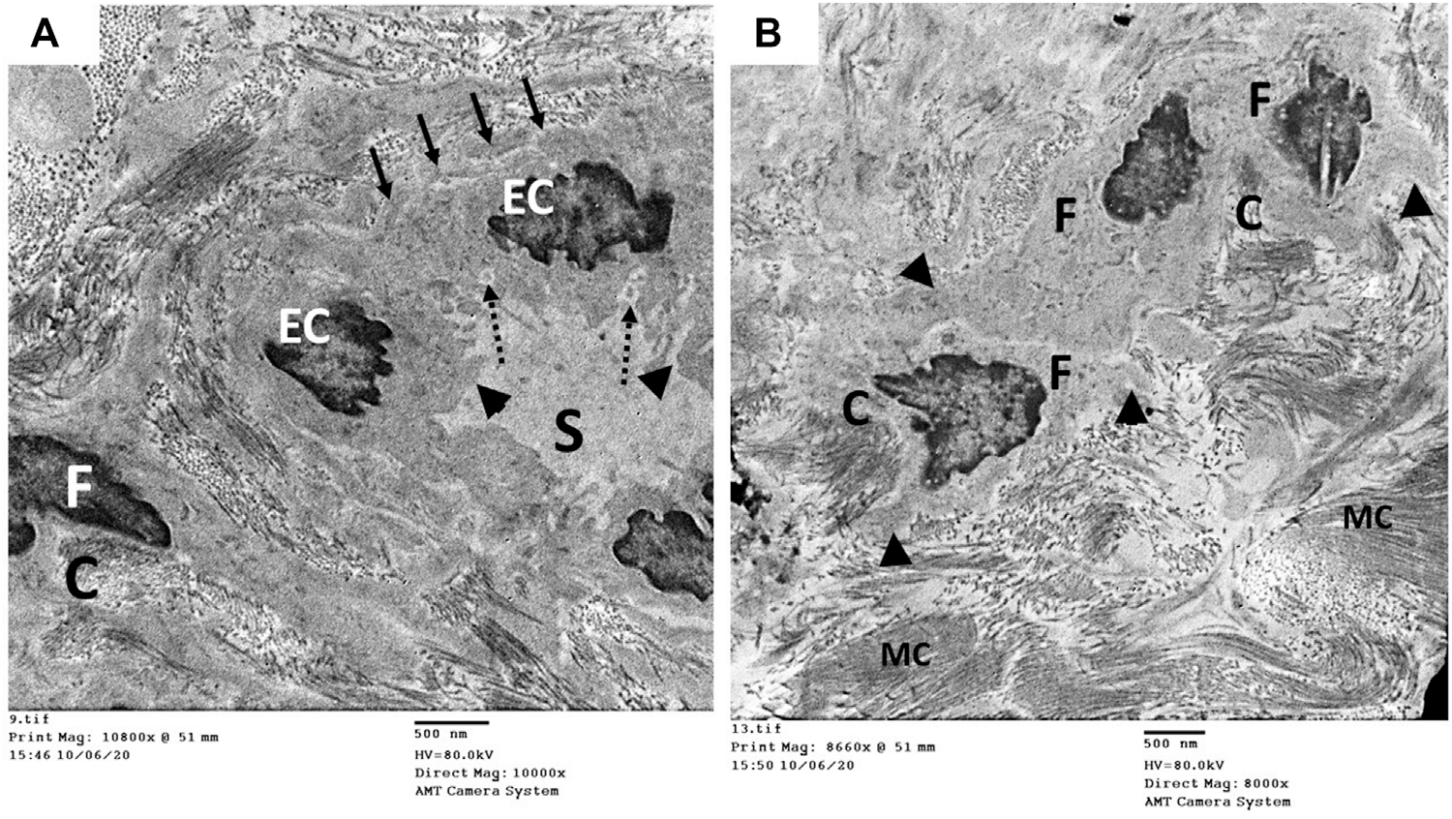
Transmission electron microscopic pictures of the corpus cavernosum (CC) of diabetic rat (group II) showing **(A)** narrow cavernous spaces (S) lined with a single layer of endothelial cells (EC) showing villi-like projection (arrowhead) into the cavernous lumen, thickened-splitted basal lamina (arrow), and many pinocytic vesicles (dashed arrow) in their cytoplasm. **(B)** The stroma of the CC showing many active fibroblasts (F) with many cytoplasmic processes (arrowhead) and surrounded by immature collagen fibers (C), and bundles of mature collagen fibers (MC) are seen at the periphery of the field.

**FIGURE 12:**
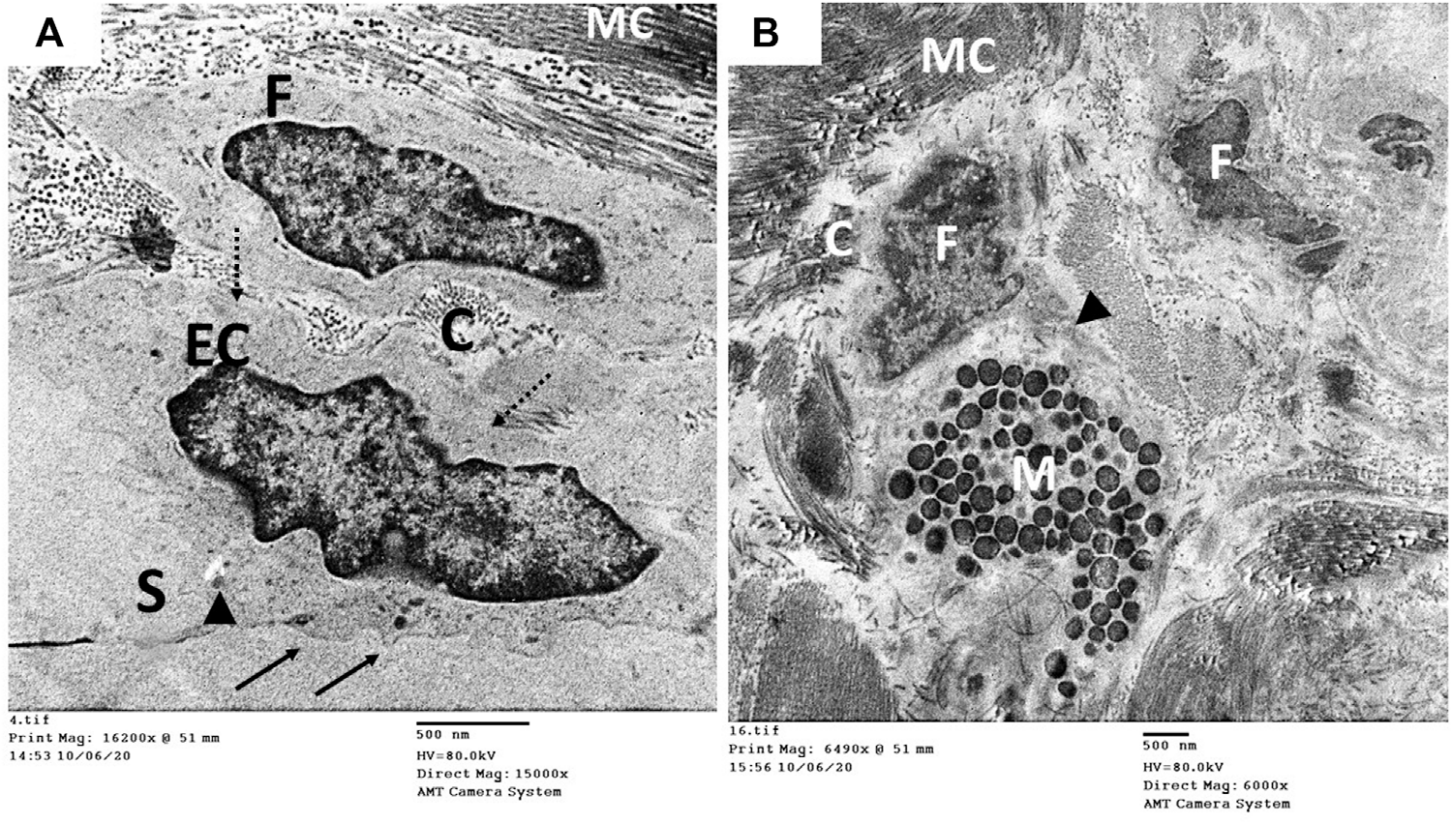
Transmission electron microscopic pictures of the CC of diabetic USC-treated group (group III) showing **(A)** one of the cavernous spaces (S) lined with endothelial cell (EC) with slightly irregular luminal surface (arrow) and moderately thickened basal lamina (dashed arrow). Few pinocytic vesicles (arrowhead) were found in its cytoplasm. One fibroblast (F) and few immature collagen fibers (C) are seen surrounding the space. **(B)** Active fibroblast (F) with cytoplasmic processes (arrowhead) surrounded by many mature collagen fibers (MC) and few immature ones (C). Mast cells (M) were noticed in several specimens of this group.

**FIGURE 13:**
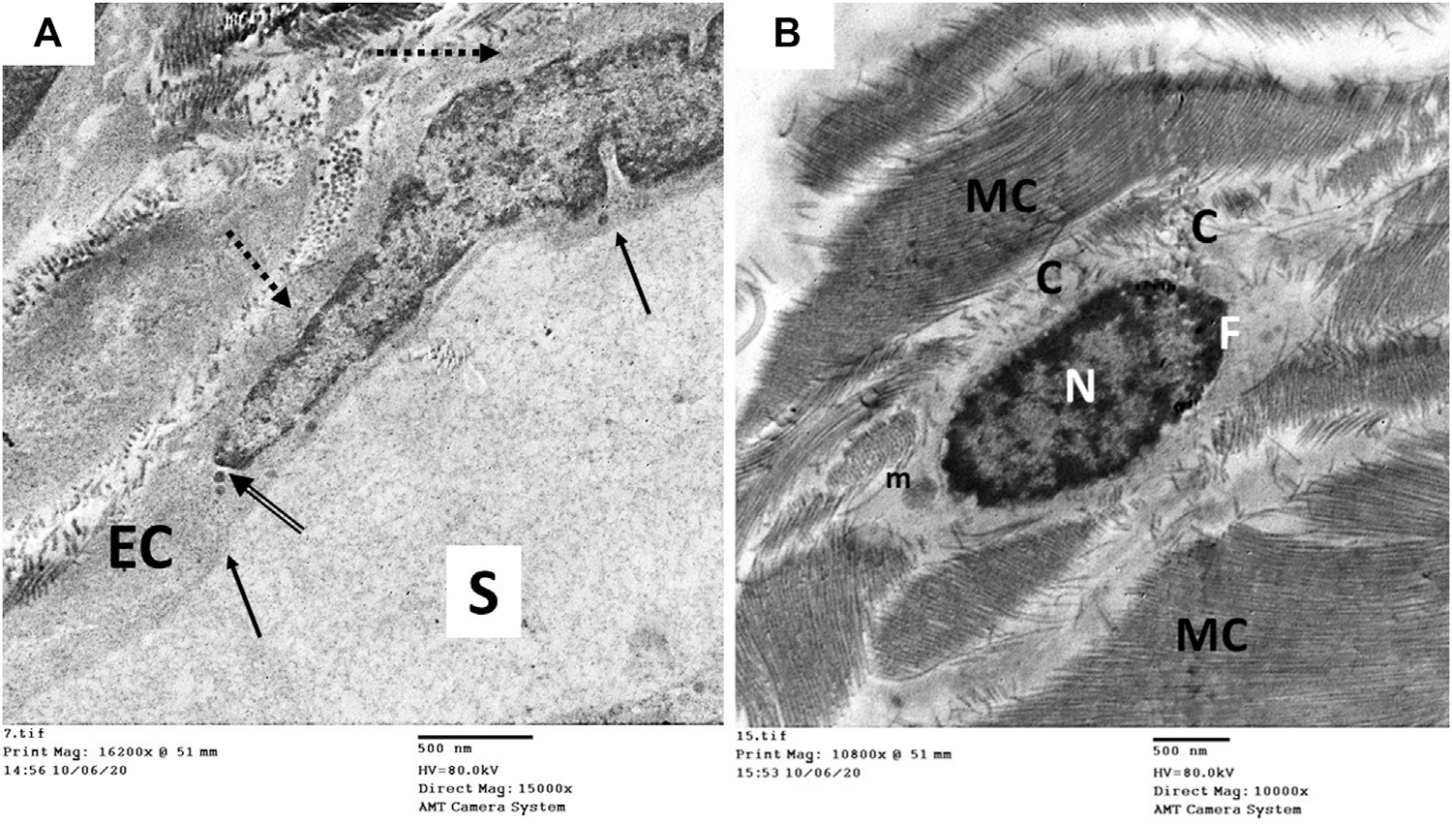
Transmission electron microscopic pictures of the CC of diabetic USC-L-treated group (group IV) showing; **(A)** one of the cavernous spaces (S) lined with endothelial cell (EC) with regular sooth luminal surface (arrow) and thin basal lamina (dashed arrow). Few pinocytic vesicles (double arrow) were found in its cytoplasm. **(B)** One fibroblast (F) with heterochromatic nucleus (N) and normal cytoplasmic organelles apart from few enlarged mitochondria (m). It exhibited no cytoplasmic processes but surrounded by many mature collagen fibers (MC) and few immature ones (C).

The ultrastructure of the CC of the control rat (group I) revealed wide cavernous spaces (CS) lined with endothelium with a thin basal lamina and smooth luminal surface. The endothelial cells also exhibited well-formed cytoplasmic organelles and almost had no pinocytic vesicles (autophagic vesicles). The CS were surrounded by bands of mature collagen fibers, having well-arranged striations; some fibroblasts were seen between these bands. These fibroblasts were supposed to be inactive due to lacking the cytoplasmic processes (Figures 10A,B). The CS of the diabetic rats (group II) were narrow due to the protrusion of the endothelium toward the lumen, which may be due to thickening, splitting of the basal lamina, and presence of villi-like projections in their luminal surface, which appeared coarse and wrinkled (Figure 11A). Many pinocytic vesicles were evident within their cytoplasm. Many active fibroblasts with multiple cytoplasmic processes were seen among the CS. They laid immature collagen fibers that appeared adjacent to their cell membrane and lost the typical striation of the mature fibers (Figure 11B). The CS of the USC-treated rats (group III) were remarkably analogous to the control ones apart from minor thickening of the basal lamina, some irregularity in the luminal surface, and the presence of few pinocytic vesicles. In addition, few active fibroblasts and immature collagen fibers were seen among the CS (Figure 12A). One-third of the animals exhibited the presence of mast cells around the collagen fibers (Figure 12B). The restoration of the endothelial structure took place in the USC-L-treated rats (group IV) regarding the regularity of the luminal surface and the basal lamina thickness (the thickening of the basal lamina was less considerable). Some pinocytic vesicles were still seen within the endothelial cytoplasm (Figure 13A). The fibroblasts appeared without cytoplasmic processes and were surrounded by mature collagen fibers (Figure 13B). Moreover, there were evident mast cells in several sections of this group.

### Presence of PKH26-Labeled USCs in the Rats’ CCs

**FIGURE 14:**
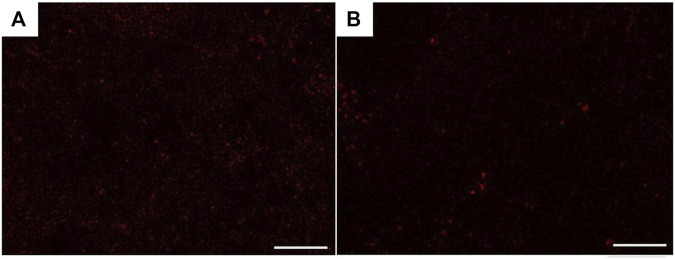
Fluorescence microscopic photomicrographs showing recruitment of PKH26-labeled USCs in the corpora cavernosa of group III rats 4 weeks **(A)** and 8 weeks **(B)** after transplantation. Scale bar: 100 μm.

The immunofluorescence examined sections of the corpora cavernosa (CCs) of USC-treated diabetic rats (group III) revealed the presence of many PKH26-labeled cells all over the CCs at the 4th week after transplantation (Figure 14A), but there was an apparent marked decrease in the number of cells by the 8th week (Figure 14B).

### Apomorphine Test as an Indicator of Erectile Dysfunction (ED)

The test was performed by the end of the 4th week after the induction of diabetes (from the first dose of STZ) and showed that 24 out of 31 diabetic rats had developed impotence. The test was repeated to all rats 8 weeks after transplantation of USCs and USC-L as an indicator of functional improvement. The animals of the control group (group I) showed no signs of erectile dysfunction (100%); only 12.5% of the diabetic rats (group II) spontaneously regained erection ability, while most of the rats in groups III and IV showed no signs of erectile dysfunction (87.5 and 93.7%), respectively.

### Copulatory Function Assessment Results

The assessment was performed on all rats in all groups just before their killing and revealed that the diabetic rats (group II) showed significantly reduced MF, IF, EL, and TE, and increased ML, IL, and PEI compared to the control rats (group I). Treatment with USCs and USCs-L significantly improves the sexual and copulatory functions in diabetic rats, evidenced by increased MF, IF, EL, and TE and decreased ML, IL, and PEI compared to diabetic rats (group II). There was no significant difference between groups III and IV regarding the measured parameters apart from El and IL (Table 1).

**TABLE 1:** Parameters of the copulatory functions (mean ± SD) of the rats of all groups.

**Measured parameter**	**Group I**	**Group II**	**Group III**	**Group IV**
Mount frequency (MF)	13.2 ± 1.2 (10)	4.5 ± 0.4* (3)	8.5 ± 0.3# (8)	10.5 ± 0.5# (8)
Intromission frequency (IF) (SCs)	9.2 ± 2 (10)	2 ± 0.01* (3)	7.4 ± 0.3# (7)	8.7 ± 0.5# (8)
Mount latency (ML) (SCs)	63.2 ± 14.3 (10)	175 ± 21* (3)	110 ± 26.6# (8)	90.8 ± 11.3# (8)
Intromission latency (IL) (SCs)	60.4 ± 13.5 (10)	190 ± 19.9**(3)	117 ± 22.1# (7)	95 ± 9.8#$ (8)
Ejaculation latency (EL)	440 ± 21.4 (10)	210.5 ± 20* (2)	311.9 ± 25# (7)	371.5 ± 32.2#$ (8)
Post-ejaculatory interval (PEI) (SCs)	430.1 ± 29.6(10)	890.1 ± 33** (2)	610.8 ± 32.2# (7)	591.5 ± 15.7# (7)
Total number of ejaculations (TE)	3.1 ± 0.01 (10)	1.3 ± 0.02* (2)	2.1 ± 0.1# (7)	2.5 ± 0.3# (8)

Latencies are measured in seconds (sec), with the other data expressed as number of occurrences. The number of animals that presented the behavior is indicated between brackets.

*p < 0.05 compared to group I.

**p < 0.001 compared to group I.

#*p* < 0.05 compared to group II.

$*p* < 0.05 compared to group III.

## Discussion

The inability of the male patient to perform a proper sexual act despite the existence of a normal sexual desire can interfere with the patient’s everyday life in several ways, including increased mental and intellectual stress, interference with normal sexual life, an increased rate of marital problems, and interpersonal social relationship issues, making erectile dysfunction (ED) a significant problem that threatens the patient’s quality of life. Although the psychological factors can play a substantial role in the etiology of (ED), this disorder is primarily attributed to organic causes in type 2 diabetic patients, such as neurological, vascular, and endothelial abnormalities (De Berardis et al., 2002).

The normal erectile function is accompanied by the relaxation of penile smooth muscle and dilatation of the arteries, leading to increased blood flow to cavernous spaces (CS) (Lee et al., 2016). Therefore, the integrity of intracavernous structures like smooth muscles, endothelium, and nerve terminals are crucially required to provide a normal erection (Albersen et al., 2011a). The interest in CS therapy strategies for erectile restoration is growing now to address the multifactorial pathogenesis of ED (Tao et al., 2017). Stem cells (SCs) therapy represents a promising new hope in this field due to their ability to act either by differentiation and direct integration within the recipient tissue or by their paracrine effect through the secretion of various growth factors and cytokines (Matz et al., 2019). In the current study, we have tested the impact of USCs in restoring the structure and the ultrastructures of corpora cavernosa (CCs) of diabetic erectile dysfunction (DED) rat model, and we have assessed the concomitant functional improvement in erection. The USC-L was also used for the same purpose of trying to confirm the USCs paracrine effect.

In this study, rats were subjected to the induction of type 2 diabetes. After which, there was a significant decrease in rats’ weight. Furthermore, the biochemical evaluation of fasting blood glucose, total cholesterol, triglycerides, and insulin tolerance test was indicative of the incidence of type 2 DM. The development of DED was confirmed using the apomorphine hydrochloride test. There was also an apparent alteration in the structure and ultrastructures of the CCs of diabetic rats and a significant deterioration in the copulatory functions of rats.

Light microscopic examination of transverse sections of the mid-shaft of diabetic rats’ penises (group II) revealed several histopathological changes. In H&E-stained sections, there was a marked narrowing of the CS, which appeared to be invaded by bands of thick collagen fibers and penile fibrosis. These findings were confirmed by Masson trichrome-stained histological sections and their quantitative analysis, which revealed that the collagen/smooth muscle ratio increased significantly in diabetic rats. The same results were obtained by (Gou et al., 2011; Ouyang et al., 2019). The immunohistochemically stained sections of the same group (group II) showed a marked decrease in the immunopositivity of α-SMA and desmin in the CC. Using α-SMA as a marker of smooth muscles (SMs) contractility is reasonable. It is the first protein found to be expressed in contractile SMs. It was proved to be downregulated in erectile dysfunction (Owens et al., 2004; Yang et al., 2014). Desmin is an intermediate filament identified in all muscle types like smooth muscle actin. It is known to have a crucial role in the differentiation and structural support of smooth muscle cells (Paulin and Li, 2004).

These findings were documented in several studies (Owens, 1995; Orr et al., 2009) which proved that the vascular SMs maintain plasticity and can change from a contractile (differentiated) to a synthetic (dedifferentiated) state. The synthetic state is characterized by a superior level of proliferation, migration, extracellular matrix production, vimentin overexpression, and decreased expression of contractile cytoskeletal proteins such as α-SMA, SM myosin heavy chain (SMMHC), and desmin. Cultured SMs of diabetic rats also exhibited significantly less contractility than those of non-diabetics and decreased expression of α-SMA, SMMHC, and desmin under hyperglycemic conditions, indicating that they could have a key role in the pathogenesis of DED (Wei et al., 2012). Moreover, Musicki and Burnett (Musicki and Burnett, 2007) demonstrated that several factors had been implicated with DED onset, including changes in SMs, collagen, and elastic fibers, which are major penile structural components that account for erection.

In the current study, electron microscopy of the diabetic group (group II) showed CS narrowing due to protrusion of the endothelium toward the lumen, which may be due to thickening and splitting of their basal lamina and marked irregularity of their luminal surface. They also exhibited pinocytotic vesicles. The same findings were noticed in the aortic endothelium of diabetic rats after 10 weeks from the induction of diabetes, along with decreased expression of their eNOS mRNA content (Lu et al., 2004). During studying the ultrastructure of the endothelium of CC in type 2 diabetic rats (Parikh et al., 2017) proved a significant decrease in their caveolae; the site of localization of endothelial NOS, which play an essential role in the penile hemodynamic required for maintaining the intracavernous pressure and erection. The pinocytotic vesicles in the endothelium of the diabetic group increased as a result of increased autophagic activity in the cells due to the presence of some metabolites resulting from hyperglycemia and active oxidative stress (Okruhlicova et al., 2005; Popov, 2010). The basal lamina thickening and splitting may alter the diffusion between the CS and the surrounding tissues and affect the cell-to-cell side talks between the endothelium and SM of the CC, which is essential for erection (Gumus et al., 2004). In diabetic patients, endothelial dysfunction appears to be a consistent finding. Hyperglycemia was proved to downregulate vascular endothelial cadherin, which in turn activates the caspase protein family, resulting in endothelial apoptosis.

Consequently, endothelial shedding as an entire cell or in the form of apoptotic endothelial microparticles takes place (Avogaro et al., 2011). This may explain the ultrastructural changes of CC endothelium in the current study. Many studies have shown a significant increase in endothelial microparticles in diabetic patients and those suffering from ED (McClung et al., 2005; La Vignera, 2011). Moreover, many active fibroblasts with multiple cytoplasmic processes were seen among the CS. They laid immature collagen fibers that appeared adjacent to their cell membrane and lost the typical striation of the mature fibers, indicating newly synthesized collagen fibers leading to penile fibrosis (Lu et al., 2004; Tao et al., 2017).

In our study, the histological sections of USCs and USC-L groups (groups III and IV) showed obvious less fibrosis and significant preservation of smooth muscle content compared to the diabetic group (group II). They exhibited apparent restoration of the CS regarding their size and structure. However, few thick collagen fibers were still seen obliterating them in the USCs group (group III). The quantitative analysis of Masson trichrome-stained sections showed that the collagen/smooth muscle ratio decreased significantly compared to the diabetic group. Equivalent results were obtained using human urine-derived stem cells either alone or genetically modified with fibroblast growth factor 2 (FGF2) (Ouyang et al., 2014). This therapeutic strategy was thought to improve the erectile functions in type 2 diabetic rats by recruiting resident cells and increasing smooth muscles’ endothelial expression and contents. Hence, restoring the smooth muscle/total collagen ratio is a key factor in the relaxation of SM and nutrition of endothelial cells in the CC. The decreased smooth muscle/total collagen ratio decreases the ability of the sinusoids to expand, resulting in veno-occlusive dysfunction (Moreland, 2000; Albersen et al., 2011b).

Marked ultrastructural repair of the CC of USCs and USC-L groups (groups III and IV) was evident compared to the diabetic group (group II) regarding the intracavernous structures and the lining endothelium of the CS. This can be explained by the proven ability of USCs to secrete proangiogenic trophic factors and immune-modulatory factors and the capability of these cells to be differentiated into endothelial cells *in vitro* (Ouyang et al., 2014).

There were multiple mast cells nearby the CS and in between the cellular component of the CCs of groups III and IV. Mast cells (MCs) are bone marrow progenitor-derived immune cells that complete their maturity in tissues. MCs are considerably affected by the local microenvironment, and they can share in numerous biological processes, including inflammation and neovascularization, through the release of several mediators and interaction with macrophages, endothelial cells, and fibroblasts (Tellechea et al., 2016). We may explain the strong presence of those cells in the treated groups (groups III and IV) by the paracrine effects of both USCs and their lysate. The paracrine mechanism of stem cell-based therapy is believed to involve the secretion of trophic factors which activate endogenous stem cells to share in tissue repair. Growth factors produced by stem cells demonstrate mitogenic, reparative, anti-apoptotic, and anti-inflammatory properties, and they also promote angiogenesis (Matz et al., 2019; Yan et al., 2020).

Regarding the functional assessment, we used the APO test (Ouyang et al., 2014) to confirm DED and to follow-up on the animals 4 and 8 weeks after transplantation. The assessment of copulatory functions was performed and showed significant improvement of all parameters in both USCs and USC-L groups compared to the DED group. Sexual behavior, sperm quantity, and quality were proved to be deteriorated even after short-term streptozotocin-induced hyperglycemia in rats (Scarano et al., 2006b) and different types of stem cell therapies were used and proved to restore the erectile functions based on measuring the intracavernous pressure in relation to the mean arterial pressure (Albersen et al., 2010; Ouyang et al., 2014; Matz et al., 2019).

In the current study, urine was used as an accessible and available source of USCs, which can be obtained via a non-invasive, simple, and low-cost approach (Ouyang et al., 2014; Zhang et al., 2014). The isolated USCs exhibited the same morphology and phenotypic character of MSCs and were proved to have high efficiency to be differentiated (Zhang et al., 2014). Zhang et al. (2008) have demonstrated that urine had few numbers of progenitor cells (range 2–7/100 ml urine). A single urine progenitor cell could form a cluster of cells and give millions of cells within a few weeks despite their few numbers. Those cells could differentiate into many cell lineages and expressed some markers of mesenchymal stem cells (CD73, CD90, and CD105), hematopoietic stem cells (CD133), and progenitor cells from different sources (epithelial and neural). Hence, they were thought to be a suitable candidate for urological tissue reconstruction. It was also stated that a single USC could expand to a large population with 60–70 population doublings, and when cultured in appropriate differentiated medium, they showed evidence of differentiation to functional smooth muscle and urothelial cells (Bharadwaj et al., 2013).

The amelioration of the erectile tissue structure and function in both treated groups in the current study could be explained in group III by either the direct engraftment of stem cells in the host tissue or by their paracrine effect. It is worth to be mentioned that the extracellular vesicles (EVs), also known as exosomes, were thought to contribute significantly to the paracrine action of stem cells. EVs can be released by many types of stem cells in the culture medium and contain genetic material, including messenger RNAs, miRNAs, and other noncoding RNAs, which could be transferred between stem cells and tissue-injured or diseased cells and then adjust the function of the recipient cells and tissues to achieve the beneficial or required action of stem cells. By binding to the 3′-untranslated regions of target messenger RNAs, miRNAs can modify gene expression post-transcriptionally (Eulalio et al., 2008). There is no sharp evidence that the stem cells lysate (SC-L) could contain EVs or parts of its genetic material. On the other hand, the SC-L was used due to having an assortment of soluble factors secreted by stem cells. Molecules such as various growth factors, hematopoietic factors, several interleukins, tumor necrosis factor-α (TNF-α), VEGF, and a variety of neurotrophins such as brain-derived neurotrophic factor (BDNF), neurturin protein, neurotrophic factor 3 (NT3), and nerve growth factor (NGF) have been identified in the secretome of SC (Salgado et al., 2010), all of which were proved to support and decrease the apoptotic index in a different component of penile tissues including endothelium, smooth muscle, and dorsal penile nerve fibers (Albersen et al., 2010).

Although there was almost no significant difference between the USCs and USC-L groups regarding the repair of penile structure and function, we could prefer the usage of USC-L, rather than USCs based on the following reasons; in our study, stem cells numbers were detected by microscopic fluorescence sections showing recruitment of PKH26-labeled USCs in the corpora cavernosa of group III rats, 4 weeks, and 8 weeks after transplantation. However, the number of USCs was significantly decreased after 8 weeks, indicating decreased survivability of stem cells after transplantation which is considered one of the limitations of their use as a cytotherapy (Yan et al., 2020). Furthermore, utilization of stem-cell free therapy by using their lysate containing rich amounts of growth factors and bioactive components or isolation of their exosomes may induce cell growth and tissue repair without taking the risk of the strange behavior of the transplanted cells in the long run or their unwanted pathways of differentiation. However, further studies aimed to identify the proteomic characteristics, the cell-free lysate’s exact components, and their mode of action is highly recommended.

## Conclusion

Both USCs and USC lysate exhibit the same DED reparative properties, and this proves the hypothesis that USCs have a paracrine effect through certain intracellular bioactive molecules and substances that help in restoring the structure and ultrastructure of CC and promote erectile functions. However, using the non-invasive technique of USC-L to cure DED may hold great promise and overcome the several disadvantages of using USCs themselves, such as decreased survivability at the site of transplantation, the unknown behavior of the transplanted cells, or the potential risk of their differentiation into undesirable/carcinogenic pathways.

## Data Availability

All data in this study are available from the corresponding author on reasonable request.

## Abbreviations

APO, apomorphine hydrochloride; α-SMA, alpha-smooth muscle actin; BM-MSCs, bone marrow-derived mesenchymal stem cells; CC, corpus cavernosum; CCs, corpora cavernosa; CM, complete media; CoS, corpus spongiosum; DED, diabetic erectile dysfunction; DMEM, Dulbecco’s modified Eagle medium; ED, erectile dysfunction; EL, ejaculation latency; FBS, fetal bovine serum; FGF2, fibroblast growth factor 2; IF, intromission frequency; IL, intromission latency; MCs, mast cells; MF, mount frequency; ML, mount latency; MSCs, mesenchymal stem cells; NO, nitric oxide; PBS, phosphate-buffered saline; PEI, post-ejaculatory interval; PS, penicillin/streptomycin; SC, stem cell; SMCs, smooth muscle cells; STZ, streptozotocin; TE, total number of ejaculation; USCs, urine-derived stem cells; USC-L, urine-derived stem cell lysate; VEGF, vascular endothelial growth factor.
