# Supplementary material for: Urine-Derived Stem Cells Versus Their Lysate in Ameliorating Erectile Dysfunction in a Rat Model of Type 2 Diabetes
Source: Front Physiol. 2022 May 10;13:854949. doi: 10.3389/fphys.2022.854949 (PMC9127444; doi:10.3389/fphys.2022.854949)
Supplement: Supplementary file 1 [file Table1.DOCX]

**Tables**

**Table 1: Parameters of copulatory function (Mean ± SD) in the rats of all groups.**

| **Measured parameter** | **Group I** | **Group II** | **Group III** | **Group IV** |
| --- | --- | --- | --- | --- |
| Mount frequencies (MF) | 13.2±1.2 (10) | 4.5±0.4* (3) | 8.5±0.3 # (8) | 10.5±0.5 # (8) |
| Intromission frequency (IF) (SCs) | 9.2±2 (10) | 2±0.01* (3) | 7.4±0.3 # (7) | 8.7±0.5 # (8) |
| Mount latency (ML) (SCs) | 63.2±14.3 (10) | 175±21* (3) | 110±26.6 # (8) | 90.8± 11.3 # (8) |
| Intromission latency (IL) (SCs) | 60.4±13.5 (10) | 190±19.9**(3) | 117±22.1 # (7) | 95±9.8 # $ (8) |
| Ejaculation latency (EL) | 440±21.4 (10) | 210.5± 20* (2) | 311.9±25 # (7) | 371.5±32.2#$(8) |
| Post-ejaculatory interval (PEI) (SCs) | 430.1±29.6(10) | 890.1±33** (2) | 610.8±32.2#(7) | 591.5±15.7# (7) |
| Total number of ejaculations (TE) | 3.1±0.01 (10) | 1.3±0.02* (2) | 2.1±0.1 # (7) | 2.5±0.3# (8) |

Latencies are measured in seconds (sec), with the other data expressed as number of occurrences. The number of animals that presented the behaviour is indicated between brackets.

*: P<0.05 compared to group I **: P<0.001 compared to group I

#: P< 0.05 compared to group II $: P< 0.05 compared to group III
