# Supplementary material for: Urine-Derived Stem Cells Versus Their Lysate in Ameliorating Erectile Dysfunction in a Rat Model of Type 2 Diabetes
Source: Front Physiol. 2022 May 10;13:854949. doi: 10.3389/fphys.2022.854949 (PMC9127444; doi:10.3389/fphys.2022.854949)
Supplement: Supplementary file 2 [file Table2.DOCX]

**Figure legends**

**Chart 1:** Panel A shows the change of body weight in control and diabetic rats. The diabetic rats showed a significant decrease of their weight 8,10 and 12 weeks after diabetes induction. Panel B showed insulin tolerance test at 8 weeks after diabetes induction. The diabetic rat showed a significant weak response to insulin. *: P<0.05

**Chart 2:** A chart showing mean values of serum glucose (A), total cholesterol and triglyceride levels (B) in all groups 8 weeks after diabetes induction. All the measured values showed a significant increase in diabetic rats (group II, III &IV) compared to control group and non-significant difference between group II, III &IV. *:P< 0.05 $: P<0.05 #: P<0.05

**Chart 3:** A bar chart showing the mean area percentage and collagen/smooth muscle ratio in the corpora cavernosa of all rats in all groups. The expression of α-SMA significantly decreased in the diabetic rats while the collage/smooth muscle ratio significantly increased in the same group. Both treated groups (group III & IV) showed almost restoration of the control values.

*: P< 0.5, indicate significance in comparison to group I.

#: P< 0.5, indicate significance in comparison to group II.

**Figure 1:** Phase contrast photomicrographs of USCs during P1 (A) and P3 (B). the cells exhibited fibroblast like appearance with short cytoplasmic processes (dashed arrow), their nuclei were vesicular with multi nucleoli (arrow). Scale bar: 100 µm. C: flowcytometric phenotypic analysis of USCs.

**Figure 2:** A histological transverse section of the penis of the control rat showing the 2 corpora cavernosa (CC) located dorsal to the corpus spongiosum (CS). UT: urethra. DV: deep dorsal vein of the penis. H&E X40. Scale bar: 500 µm

**Figure 3:** Histological transverse sections of the penis of all rats in all groups. A: corpus cavernosum in control group appeared with wide cavernous spaces and formed by smooth muscles and collagen fibres. B: showed narrowing of the cavernous spaces while C&D showed almost restoration of them in group III and IV, respectively. S: cavernous spaces. Arrow: smooth muscles. Asterisk: collagen fibres. H&E X100 scale bar: 100 µm

**Figure 4:** Histological transverse sections of the penis of all rats in all groups stained with Masson Trichrome stain, the blue and the red colour indicate collagen and smooth muscle, respectively. The diabetic rat showed increase collagen expression (B) in comparison to the control rats (A). Collagen expression in group III and IV greatly resembled its expression in the control group. Masson Trichrome X400 scale bar: 50 µm.

**Figure 5:** Histological transverse sections of the penis of all rats in all groups immunohistochemically stained with anti-α-SMA antibody showing diminution its expression in group II (B) in comparison to the control group (A) and restoration of its expression in group III and IV (C&D, respectively). X400 scale bar: 50µm.

**Figure 6:** Transmission electron microscopic pictures of the control rat corpus cavernosum showing: A) wide cavernous spaces (S) lined with endothelium (EC) and surrounded by mature collagen fibres (C) and fibroblasts (F). B) the lining endothelium showing well identified nucleus (N), narrow basal lamina (arrow) and smooth cavernous surface (arrowhead) but lacking the pinocytic vesicles in the cytoplasm.

**Figure 7:** Transmission electron microscopic pictures of the diabetic rat (group II) corpus cavernosum showing: A) narrow cavernous spaces (S) lined by a single layer of endothelium (EC) that showing villi-like projection (arrowhead) into the cavernous lumen, thickened-splitted basal lamina (arrow) and many pinocytic vesicles (dashed arrow) in their cytoplasm. B) The stroma of the CC showing many active fibroblasts (F) with many cytoplasmic processes (arrowhead) and surrounded by immature collagen fibres (C) bundles of mature collagen fibres (MC) are seen at the periphery of the field.

**Figure 8:** Transmission electron microscopic pictures of the diabetic rat treated with USCs (group III) corpus cavernosum showing: A) one of the cavernous spaces (S) lined with endothelial cell (EC) with slightly irregular luminal surface (arrow) and moderately thickened basal lamina (dashed arrow). Few pinocytic vesicles (arrowhead) were found in its cytoplasm. One fibroblast (F) and few immature collagen fibres (C) are seen surrounding the space. B) active fibroblast (F) with cytoplasmic processes (arrowhead) surrounded by many mature collagen fibres (MC) and few immature ones (C). few mast cells (M) were noticed in many specimens.

**figure 9:** Transmission electron microscopic pictures of the diabetic rat treated with USCs-L (group IV) corpus cavernosum showing: A) one of the cavernous spaces (S) lined with endothelial cell (EC) with regular sooth luminal surface (arrow) and thin basal lamina (dashed arrow). Few pinocytic vesicles (double arrow) were found in its cytoplasm. B) one fibroblast (F) with heterochromatic nucleus (N) and normal cytoplasmic organelles apart from few enlarged mitochondria (m). it exhibited no cytoplasmic processes but surrounded by many mature collagen fibres (MC) and few immature ones (C).

**Figure 10:** Fluorescence microscopic photomicrographs showing recruitment of PKH26-labeled USCs in the corpora cavernosa of group III rats, 4 (A) and 8 (B) weeks after transplantation. Scale bar: 100 µm.
